# Giardiasis Outbreaks — United States, 2012–2017

**DOI:** 10.15585/mmwr.mm7009a2

**Published:** 2021-03-05

**Authors:** Erin E. Conners, Allison D. Miller, Neha Balachandran, Brittany M. Robinson, Katharine M. Benedict

**Affiliations:** ^1^Division of Foodborne, Waterborne, and Environmental Diseases, National Center for Emerging and Zoonotic Infectious Diseases, CDC; ^2^Epidemic Intelligence Service, CDC; ^3^Division of Viral Diseases, National Center for Immunization and Respiratory Diseases, CDC.

Giardiasis is a diarrheal disease caused by the parasite *Giardia duodenalis*, the most common cause of intestinal parasite infections in the United States. Transmission occurs when *Giardia* cysts spread from feces to water, food, surfaces, or skin and are then ingested. Illness is characterized by gastrointestinal symptoms, including diarrhea, abdominal cramps, greasy stools, bloating or gas, nausea, vomiting, weight loss, and dehydration. Approximately 50% of infections are asymptomatic (*1*,*2*). Most symptomatic *Giardia* infections are self-limited in duration; however, some persons might experience a reoccurrence of symptoms or develop long-term complications (*3*). During 2012–2017, public health officials from 26 states reported 111 giardiasis outbreaks (760 cases) to the National Outbreak Reporting System (NORS). Three main modes of transmission for these outbreaks were identified: water exposure in 29 (26%) outbreaks, person-to-person contact in 28 (25%) outbreaks, and contaminated food in six (5%) outbreaks. A single transmission mode could not be determined in 48 (43%) of the outbreaks. Private residences and child care facilities were the most common settings of outbreaks for all the transmission modes combined. To prevent and control giardiasis outbreaks, CDC recommends prompt diagnosis, maintaining good hand hygiene, cleaning and disinfecting home environments and child care facilities, and monitoring water quality in private wells.

A giardiasis outbreak is defined as the occurrence of two or more cases of illness epidemiologically linked to a common exposure (*1*). Health department officials from across the United States (state, local, and District of Columbia), U.S. territories,* and freely associated states^†^ voluntarily report outbreaks to NORS. This study included giardiasis outbreak reports submitted to NORS by December 30, 2019 and data reported during 2012–2017 (the year of the earliest case illness onset date through the most recent year for which data were available). NORS data summarized in this study include primary case counts, hospitalizations, and deaths; transmission mode; exposures and settings; and earliest onset date. Negative binomial regression analysis was conducted to assess for annual trends in outbreak counts using SAS (version 9.4; SAS Institute). This activity was reviewed by CDC and conducted consistent with applicable federal law and CDC policy.^§^

During 2012–2017, public health officials from 26 states reported 111 giardiasis outbreaks with 760 primary cases, 28 hospitalizations, 48 emergency department visits, and no deaths. Among the 703 cases with available data, 370 (53%) persons were male and 333 (47%) persons were female. Pennsylvania reported the largest number of outbreaks with 44 (40%), followed by Minnesota with 11 (10%); no other state reported >10 outbreaks (Figure 1). There was no significant trend in giardiasis outbreaks by year (χ^2^ = 0.67, p = 0.98) (Figure 2).

**FIGURE 1:**
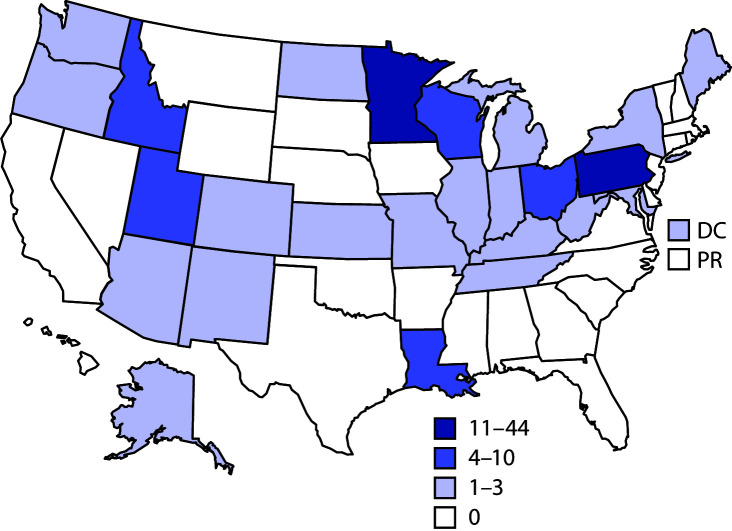
Reported giardiasis outbreaks (N = 111), by jurisdiction — National Outbreak Reporting System, United States, 2012–2017* **Abbreviations**: DC = District of Columbia; PR = Puerto Rico. * These numbers are dependent on reporting requirements and public health capacity, which vary across jurisdictions and do not necessarily indicate the actual occurrence of giardiasis outbreaks in a given jurisdiction.

**FIGURE 2:**
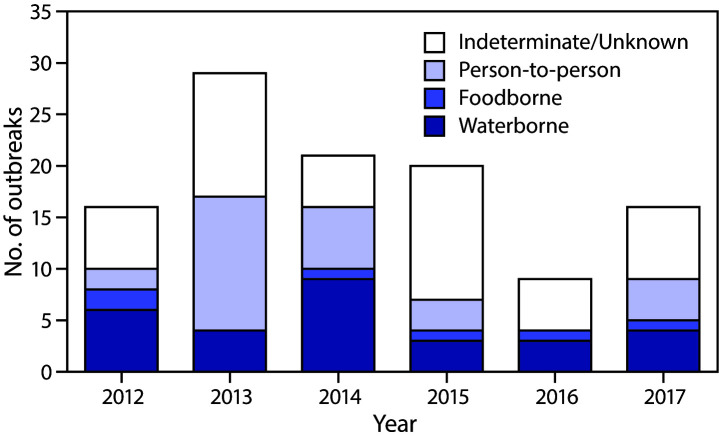
Reported giardiasis outbreaks (N = 111), by mode of transmission* and year of earliest illness onset date — United States, 2012–2017 * Transmission modes were categorized as follows: indeterminate/unknown if evidence to implicate one specific primary mode of transmission was insufficient; person-to-person if transmission occurred from direct contact with an infected person, their body fluids, or by contact with the local environment where the exposed person was present; foodborne if transmitted through consumption of contaminated food or non-water beverages; waterborne if transmission occurred via ingestion, inhalation, contact, or another exposure to water (e.g., treated or untreated recreational water, drinking water [including bottled water], or an environmental or indeterminate water source). There were no outbreaks attributed to animal contact or environmental contamination other than food and water (https://www.cdc.gov/nors/forms.html).

Among 29 (26%) waterborne outbreaks (370 cases), exposure sources included tap water systems (e.g., municipal systems or private wells) in nine outbreaks, outdoor freshwater consumption in seven outbreaks, treated recreational water in five outbreaks, untreated recreational water in four outbreaks, and “other” in four outbreaks (Table). Reported settings for waterborne outbreaks included 12 (41%) outdoor areas (e.g., parks and forests) five (17%) private residences, four (14%) camps or cabins, three (10%) community/municipality settings, three (10%) unknown, and two (7%) other settings. Person-to-person transmission was the primary mode identified in 28 (25%) outbreaks, resulting in 129 cases. The primary exposure settings for these outbreaks were 14 (50%) private residences and 12 (43%) child care facilities (Table). Among the 14 settings in private homes, nine (64%) were in households with children aged ≤5 years; two (14%) were in homes with only adults. Among the six (5%) foodborne outbreaks, all foods associated with the five known food exposures were eaten raw or with minimal or no processing. No outbreaks were attributed to animal contact or environmental contamination other than food and water (i.e., contact with objects or surfaces with *Giardia*). Among all 111 outbreaks, 48 (43%) had an indeterminate or unknown transmission mode, meaning that there was insufficient evidence to implicate one specific primary mode of transmission; 33 (69%) of these outbreaks occurred in private residences (Table).

**TABLE:** Reported giardiasis outbreaks (N = 111), by mode of transmission and exposure — United States, 2012–2017

Transmission mode	No. (%)	
	Outbreaks	Cases
All modes	111 (100)	760 (100)
**Waterborne (exposure source)**	**29 (26)**	**370 (49)**
Tap water systems*	9	94
Recreational water		
Treated (e.g., pool)	5	19
Untreated (e.g., lake)	4	135
Outdoor freshwater (drinking source)	7	103
Other^†^	4	19
**Person-to-person (exposure setting)**	**28 (25)**	**129 (17)**
Private home/residence	14	47
Child care facilities	12	49
School/College/University	2	33
**Foodborne (food vehicle)** ^§^	**6 (5)**	**97** (**13)**
Oysters, raw	3	14
Whole milk, unpasteurized	1	38
Mixed green salad	1	25
Undetermined^¶^	1	20
**Indeterminate/Unknown** (exposure setting)**	**48 (43)**	**164 (22)**
Private home/residence	33	93
Child care facilities	2	25
Other^††^	5	17
Undetermined^§§^	8	29

* Includes municipal systems and private wells.

^†^ Other waterborne outbreaks involved the following exposures: undetermined exposure to outdoor freshwater (two), sewage effluent (one), and undetermined exposure to water (one).

^§^ Includes confirmed and suspected food vehicles.

^¶^ Foodborne outbreaks were categorized as undetermined if specific food vehicle was not identified or reported.

** Outbreaks were categorized as having an indeterminate/unknown transmission mode if there was insufficient evidence to implicate one specific primary mode of transmission.

^††^ Indeterminate/unknown outbreaks categorized as “other” involved the following settings: communitywide (one), fair/festival (one), prison/detention center (one), resort (one), workplace (one).

^§§^ Indeterminate/unknown outbreaks were categorized as undetermined if specific setting was not identified or reported.

## Discussion

Among giardiasis outbreaks transmitted person-to-person, and by waterborne and indeterminate transmission modes, 50% (52 of 105) occurred in private residences. Patients with giardiasis might be infectious for several weeks, and ingestion of as few as 10 cysts can cause disease, making good hygiene a critical component of preventing further disease spread (*4*). After a giardiasis diagnosis, prevention messaging to patients and their household members should include the importance of good hand hygiene practices, especially before preparing food or eating and after using the bathroom or changing diapers, as well as cleaning and disinfecting the home environment. Beyond the home, these hygiene recommendations also apply to child care facilities, another frequently reported setting for giardiasis outbreaks. CDC also recommends that persons with *Giardia* infection abstain from sexual activity for at least 2 weeks after diarrhea has resolved, because *Giardia* organisms can be transmitted through sexual contact (*5*).

Households that use tap water from a private well should be advised to have their water tested at least once a year to monitor water quality and for any contaminant of concern based on local conditions (e.g., septic system overuse, nearby wastewater discharges, agricultural or industrial runoff, or contaminants detected in neighbors’ wells).

Reported waterborne outbreaks of giardiasis were associated primarily with exposures to outdoor freshwater sources, either through drinking or recreational water use. Because drinking water directly from these sources is not regulated, health promotional materials should include information on the health risks of consuming water from outdoor freshwater sources and guidance on how to adequately treat water before consumption. Untreated water or ice from lakes, rivers, streams, ponds, or shallow wells should not be consumed. Because the infectious dose of *Giardia* is small and *Giardia* cysts are immediately infectious and moderately resistant to chlorine disinfection, CDC recommends that persons should not swim if sick with diarrhea and for at least 2 weeks after diarrhea has resolved.^¶^

Prompt diagnosis and treatment of giardiasis can also prevent further spread. Many patients endure symptoms long before they receive a diagnosis. For example, in a U.S. study of insurance claims data, one half of giardiasis patients required three or more office visits before the diagnosis was made; in >20% of patients, a giardiasis diagnostic code was not recorded until >30 days after the initial visit for gastrointestinal symptoms (*6*). Because *Giardia* organisms are excreted intermittently in feces, sensitivity of microscopy with the direct fluorescent antibody test (the standard for diagnosis) can be increased by collecting and testing three stool specimens from patients on separate days (*7*,*8*). If available, molecular-based gastrointestinal panel assays that include *Giardia* as a target pathogen might also be used for diagnosis (*9*). Nitroimidazoles, including metronidazole and tinidazole, are efficacious first-line drugs available in the United States (*10*).

The Council for State and Territorial Epidemiologists changed the confirmed case definition for giardiasis in 2011 from requiring only laboratory confirmation to requiring laboratory confirmation and meeting the clinical description for illness (*1*). Despite this stricter definition, there was no statistically significant decline in the number of reported outbreaks during 2012–2017.

The findings in this report are subject to at least two limitations. First, the number of outbreaks and their associated cases reported to NORS are likely underestimates, as resources for investigating and reporting outbreaks vary among states. Less than one half of states reported any giardiasis outbreak. It is unclear whether the geographic spread of outbreaks represents the actual underlying distribution, or whether it is a surveillance artifact. Second, attribution to a single transmission mode was not always possible. Identifying a single transmission mode might be particularly challenging among close household contacts who have overlapping exposures.

Results from this study suggest a need to focus prevention messages on the settings of giardiasis outbreaks, rather than on a single transmission mode, as nearly one half of reported outbreaks had an unknown mode of transmission. In view of these results, giardiasis prevention and control initiatives and health materials should promote prompt diagnosis, maintaining good hand hygiene, cleaning and disinfecting home environments and child care facilities, and monitoring water quality in private wells.

**Summary**

**What is already known about this topic?**

Giardiasis is a diarrheal disease caused by the parasite *Giardia duodenalis*, the most common cause of intestinal parasite infections in the United States.

**What is added by this report?**

During 2012–2017, public health officials from 26 states reported 111 giardiasis outbreaks involving 760 cases. Leading causes of outbreaks were waterborne and person-to-person exposures. Private residences and child care facilities were the most common settings of giardiasis outbreaks across all transmission modes.

**What are the implications for public health practice?**

To prevent and control giardiasis outbreaks, CDC recommends prompt diagnosis, maintaining good hand hygiene, cleaning and disinfecting home environments and child care facilities, and monitoring water quality in private wells.

## Footnotes

* American Samoa, Northern Mariana Islands, Guam, Puerto Rico, and U.S. Virgin Islands.

† Federated States of Micronesia, Marshall Islands, and Palau.

§ 45 C.F.R. part 46.102(l)(2), 21 C.F.R. part 56; 42 U.S.C. Sect. 241(d); 5 U.S.C. Sect. 552a; 44 U.S.C. Sect. 3501 et seq.

¶
https://www.cdc.gov/healthywater/swimming/swimmers/rwi/diarrheal-illness.html
